# White matter connectivity in resilience in a general population sample of 12,516 individuals

**DOI:** 10.1016/j.nsa.2023.101130

**Published:** 2023-07-27

**Authors:** B.S. de Vries, N.J.A. van der Wee, S.J.A. van der Werff

**Affiliations:** a Department of Psychiatry, Leiden University Medical Center, Leiden, the Netherlands; b Leiden Institute for Brain and Cognition, Leiden, the Netherlands

**Keywords:** Resilience, Psychological”, “Diffusion tensor imaging”, “Psychological trauma”

## Abstract

Neurobiological correlates of resilience to stress or trauma have not been investigated extensively. Studies on white matter connectivity in resilience, in particular, have been few and far between. This explorative study included 12,516 participants from the UK Biobank to investigate white matter connectivity in resilience in 13 selected regions of interest (ROIs). The data for this cross-sectional cohort study were retrieved from UK Biobank, a large-scale biomedical database and research resource. The study included 40-69-year-old women and men, as enrolled by UK Biobank, (N ​= ​12,516) divided into three groups: a trauma-exposed, healthy (resilient) group, a trauma-exposed, mentally ill (vulnerable) group and a nonexposed, healthy (control) group. The primary outcome measures consisted of mean fractional anisotropy values in the following ROIs: cingulate gyrus, cingulum hippocampus, superior fronto-occipital fasciculus, uncinate fasciculus, corpus callosum body, genu, splenium, and tapetum. Group differences in fractional anisotropy were assessed using a one-way analysis of variance. This study did not find correlates of resilience in the investigated ROIs. The results indicate that there is no association between resilience and white matter connectivity in the selected ROIs in this large population sample, which is in contrast with previous studies on morphometric and functional correlates of resilience. To corroborate this finding, the authors recommend collection of longitudinal data and utilization of voxelwise analysis, if possible, to improve sensitivity. The authors also suggest a whole-brain analysis in a similar study population to investigate other white matter tracts that might be implicated in resilience.

## 1 Introduction

In the preceding decades, researchers in the field of psychiatry and neuroscience have become increasingly interested in the neurobiology related to the concept of resilience. Resilience is often defined as a dynamic mechanism that consists of positive adaptation within the context of significant adversity, and furthermore, from a more psychobiological standpoint, as short- and long-term adaptive processes that reduce allostatic load (Charney, 2004; Cicchetti and Rogosch, 2009; Curtis and Cicchetti, 2003; van der Werff et al., 2013). In studies on resilience, it is often more plainly operationalized as the absence of psychopathology after experiencing a traumatic event. Studying resilience to trauma could contribute to the development of new treatment and prevention strategies for individuals at high risk of traumatic exposure. Importantly, while resilience has already been studied extensively in its psychological context, neurobiological research on resilience, in particular research on neural mechanisms, is lagging behind. The main focus of neuroimaging of resilience so far has been on morphometric and functional correlates of resilience, using magnetic resonance imaging (van der Werff et al., 2013). Contrastingly, white matter integrity in resilience has, to our knowledge, been studied to a limited extent and only insmall sample sizes, even though the interconnectivity of brain regions through white matter tracts could very well be implicated in resilience.

So far, two animal studies have investigated white matter connectivity in resilience models. One study showed lower fractional anisotropy (FA), a measure of white matter integrity, in the hippocampus, hypothalamus, nucleus accumbens, cingulate cortex and amygdala for resilient (social stress exposed, normal behaving) mice compared to vulnerable (social stress exposed, socially avoidant) mice (Anacker et al., 2016). In contrast, another study, reported no significant FA differences between resilient, control (nonexposed, normal behaving) and vulnerable mice (Liu et al., 2018).

In humans, studies on white matter integrity in resilience are scarce and of heterogeneous designs. The available data consists mostly of studies focused on small samples of high-risk individuals such as first responders and military personnel. Studies typically only include patients with post-traumatic stress disorder (PTSD) in the group with mental illness and trauma exposure, even though a traumatic event is also known to be a precipitating factor for many other psychiatric disorders (Dohrenwend, 1998; Koenigs et al., 2008; Kuo et al., 2012; van der Werff et al., 2017). Importantly, the data is scarce to such a degree that researchers interested in white matter correlates of resilience have had to make do with studies on vulnerability. These studies usually compare a healthy, trauma-exposed (resilient) group to a mentally ill, trauma-exposed (vulnerable group). This is far from ideal, since, without a healthy, nonexposed (control) group, it is not possible to distinguish the neural correlates of resilience and those of mental illness (van der Werff et al., 2013).

Some studies have used a design with such a control group. A study by O'Doherty et al. (2018) (N ​= ​75) found lower FA in the uncinate fasciculus (UF), cingulum cingulate gyrus (CG), superior longitudinal fasciculus, corpus callosum body (CCB), genu (CCG) and splenium (CCS) in a trauma-exposed, healthy group and in a PTSD group compared to a healthy, non-exposed control group. Another study by van der Werff et al. (2017) (N ​= ​81) investigated white matter connectivity in highly resilient police officers with whole brain analysis and found an increase in structural connectivity in the corticopontine tract in the resilient group. A larger sized study by the PGC-ENIGMA PTSD consortium (N ​= ​3047), though not designed specifically to detect correlates of resilience, showed higher FA in the corpus callosum tapetum (CCT) and superior fronto-occipital fasciculus (SFOF) for the resilient (trauma-exposed, non PTSD) group (N ​= ​113) compared to the vulnerable (trauma-exposed, PTSD) group (N ​= ​2704) and lower FA in CCT, CCS and fornix or stria terminalis in the resilient group compared to nonexposed controls (N ​= ​180) (Dennis et al., 2019). All in all, white matter tracts most likely to be implicated in resilience, as evidenced by what has been published so far on resilience and vulnerability, are the CG, cingulum hippocampus (CH), SFOF, UF, CCB, CCG, CCS and CCT (Abe et al., 2006; Aschbacher et al., 2018; Dennis et al., 2019; Fani et al., 2012; Kim et al., 2006; Koch et al., 2017; Li et al., 2016; Meng et al., 2018; O'Doherty et al., 2018; van der Werff et al., 2013; van der Werff et al., 2017; Wang et al., 2010).

To investigate integrity of these white matter tracts in the context of resilience in a large population sample, we conducted a cross-sectional cohort study with diffusion tensor imaging (DTI) scans available from UK Biobank, a large-scale biomedical database and research resource based in the United Kingdom. The resource, which includes lifestyle and health information, blood samples, heart and brain scans and genetic data of 500,000 volunteer participants between 40 and 69 years old, is globally accessible for health-related research that is in the public interest.

The aim of this study was to investigate mean FA values in the CG, CH, SFOF, UF, CCB, CCG, CCS and CCT to determine white matter integrity in resilient brains compared to vulnerable and control brains. Due to the scarcity of the available evidence, this study was explorative in nature and therefore no hypothesis was defined.

## 2 Experimental procedures

### 2.1 Participants

We received approval from UK Biobank to conduct this study under project number 31102. All data and materials, including information on the informed consent process, are available via UK Biobank. UK Biobank has approval from the North West Multi-center Research Ethics Committee (MREC). The study was carried out in accordance with *The Code of Ethics of the World Medical Association (Declaration of Helsinki) for experiments involving humans* and the *Uniform Requirements for manuscripts submitted to Biomedical journals*.

The participants were drawn from the source population of the UK Biobank, which consists of 40-69-year-old volunteers residing in the United Kingdom (Sudlow et al., 2015). Participants were requested to attend one or more assessments, including a general assessment that included several questionnaires, and an imaging assessment. All participants were right-handed and had completed the imaging assessment and the questionnaire on mental health. We excluded patients that reported mental illnesses that are known to have a strong genetic component. We also excluded patients that reported somatic diagnoses that would impact brain structure or connectivity. Thus, exclusion criteria were: past or current diagnosis of a psychotic disorder, bipolar disorder, obsessive compulsive disorder, any phobia except for agoraphobia, attention deficit or attention deficit and hyperactivity disorder or an autism spectrum disorder; diagnosis of any neurological injury, illness or a neurological developmental or chronic problem; diagnosis of an endocrinological problem that that could impact brain structure or connectivity; withdrawal from the UK Biobank study, as communicated through UK Biobank.

### 2.2 Group selection

Subjects were divided into three groups: a resilient group (having experienced a traumatic event, and reporting no life-time mental illness), a vulnerable group (having experienced a traumatic event and reporting a diagnosis during their lifetime of one or more mental illnesses) and a control group (having not experienced a traumatic event, and reporting no life-time mental illness). We excluded the group that scored negative on trauma exposure and positive on mental illness.

For assessment of trauma exposure, four binomial variables were used, in addition to three ordinal variables that used a five-point scale (never, rarely, sometimes, often, very often). Physical and sexual abuse as a child as well as domestic abuse by an (ex-)partner in adulthood were considered traumatic events if the participant reported to have experienced this sometimes, often or very often. Having experienced a life-threatening accident, sexual assault or a physically violent crime and having witnessed sudden violent death were also classified as traumatic events.

Mental illness, or a history thereof, was defined as having reported one or more mental health problems ever diagnosed by a professional or having ever been addicted to any substance (excluding caffeine and tobacco) or behavior (such as gambling).

At this time, in order to make a clear distinction between the resilient and the control group, we excluded participants from the control group if they had experienced trauma infrequently, or had experienced a type of trauma that we did not include in our assessment of trauma exposure. Thus, we excluded participants from the control group that had rarely experienced physical or sexual abuse of a child or domestic abuse by an (ex-)partner in adulthood, as well as controls that had rarely, sometimes, often or very often experienced sexual interference by an (ex-)partner without consent, had felt hated by a family member as a child or had been belittled by an (ex-)partner as an adult. Lastly, we excluded controls if they had been involved in combat or if they had been diagnosed with a life-threatening illness.

### 2.3 Questionnaires

Information derived from the questionnaires on mental health, medical conditions, early life factors and education was used for inclusion and the assessment of education level. The study demographics of sex, age and socio-economic status were derived from the central registry of participants. Socio-economic status was derived from the Townsend deprivation index. The mental health questionnaire was also used to classify a participant as resilient, control or vulnerable. To determine highest attained level of education by the participants, reported qualifications were divided into three categories. Basic education consisted of primary education or no school education, secondary education was classified as a high school education, and tertiary education consisted of university education or a vocational degree.

At the UK Biobank assessment center, questionnaires were, whenever possible, administered using a computer on which participants could work independently. For data fields that required detailed questioning, such as taking a medical history, computer-assisted interviews were held by members of staff. Participants were also requested to gather information on medical history beforehand to improve recall (Sudlow et al., 2015).

### 2.4 Magnetic resonance imaging procedures

A Siemens Skyra 3T scanner with a standard Siemens 32-channel RF receive head coil was used for the imaging assessments. The DTI images were acquired using a spin-echo echo-planar sequence with voxel dimensions of 2 ​× ​2 ​× ​2 mm. Five T2-weighted (b ​= ​0 ​s/mm^2^) baseline volumes, 3 blip-reversed T2-weighted (b ​= ​0 ​s/mm^2^) baseline volumes, 50 ​b ​= ​1000 ​s/mm^2^ and 50 ​b ​= ​2000 ​s/mm^2^ diffusion-weighted volumes were acquired with in total 100 distinct diffusion-encoding directions (Alfaro-Almagro et al., 2018).

All preprocessing and quality checks were performed by the UK Biobank team. A basic automated quality check was performed to remove unusable or corrupted data. The images were corrected for eddy currents and head motion, and outlier slices were corrected, using the Eddy tool (Andersson and Sotiropoulos, 2016). The DTI outputs of FA, mean diffusivity (MD), axial diffusivity (AD) and radial diffusivity (RD) were created using the DTI fitting tool DTIFIT. Tract-based spatial statistics-derived measures were computed by averaging the skeletonized images of each DTI map within a set of 48 standard-space tract masks defined by the Johns Hopkins University White Matter Atlas (ICBM-DTI-81), which resulted in the predefined mean FA white matter tracts that were used for this study (Alfaro-Almagro et al., 2018; Mori et al., 2005; Smith et al., 2006; Wakana et al., 2007).

### 2.5 Statistics

To compare study demographics between groups, we used a chi-square test, a one-way analysis of variance (ANOVA) and a Kruskall-Wallis test for the categorical, continuous and ordinal variables, respectively. Group differences in white matter FA for each region of interest (ROI) were assessed using a one-way ANOVA. Sex and age were added as covariates, unless the difference from the mean for either of these variables was more than 5% for any one of the groups. In that case, a mediation analysis was performed instead of adding the variable as a covariate.

If the ANOVA was significant, we conducted three one-tailed T-tests comparing FA means between the three groups. If applicable, covariates were added. In the event that such a T-test between two groups proved significant, MD, RD and AD means for that region of interest were compared between the two groups to facilitate further interpretation. For this we used one-tailed T-tests, seeing as the direction of the possible association could be derived from the FA results. To represent effect size, we used Cohen’s *d*.

To control for multiple testing, we corrected the results of the statistical tests with the adjusted Bonferroni correction (Dennis et al., 2019; Li and Ji, 2005). This method included a calculation of the effective number of independent tests (M_eff_) and took into account the assumption that the statistical tests were not independent, since the studied ROIs were interconnected white matter tracts. For the FA ROIs, the correlation coefficient ranged between 0.101 and 0.813. We calculated a M_eff_ of 9, which yielded a threshold for significance of 0.0057.

Based on the results, it was decided to perform a post hoc analysis using a definition of trauma exposure focused on childhood trauma, which was expected to have the largest impact on connectivity. Two variations were used: in the wider definition, the responses “sometimes”, “often” and “very often” for physical or sexual abuse as a child were included, in the narrower definition, only “often” and “very often” were included. It was expected that using these definitions of severe, early trauma, resilient and vulnerable brains would show altered connectivity patterns.

## 3 Results

We received mental health data and DTI data on 18,589 participants, of which 16,554 were right-handed. After somatic and psychiatric exclusion criteria, 14,586 participants remained, of which 11,184 participants did not report a history of mental illness. Of this number, 3070 participants had experienced a traumatic event and thus were classified as resilient. After exclusion criteria for controls in the remaining 8114 participants, 7577 controls were included. Of the 3402 participants that reported a history of mental illness, 1869 participants had experienced a traumatic event and thus were classified as vulnerable.

Study demographics for the resilient, control and vulnerable groups are displayed in Table 1. The proportion of women was significantly higher in the vulnerable group (61.9%) as compared to the resilient (44.1%) and control group (50.2%, p ​< ​0.001). Furthermore, the vulnerable group was on average slightly younger (61.1 years old) compared to the resilient and control groups (63.0 and 63.2 years respectively, p ​< ​0.001), though this did not seem a relevant difference in age, given also the large sample size. Tertiary education was more prevalent in the resilient and vulnerable groups (p ​< ​0.001). Townsend deprivation index was higher in the vulnerable group, indicating lower socio-economic status. However, given the large range, this difference was not deemed relevant. Age was added as a covariate to remove effects of aging on the brain from the results. Townsend deprivation index was not, as socio-economic status is highly associated with vulnerability, both as a risk factor for and as a consequence of vulnerability. We also performed a mediation analysis for the effect of sex on the results. We did not correct for level of education because we hypothesized that level of education, as an index of intelligence, would impact white matter connectivity. Studies have shown that resilient individuals more often pursue higher education, thus level of education would be in the causal pathway for the association between resilience and white matter connectivity (Werner and Smith, 1992).

**Table 1:** Baseline characteristics.

		Resilient (N ​= ​3070)		Control (N ​= ​7577)		Vulnerable (N ​= ​1869)		Total (N ​= ​12,516)		*p*
		*N*	*%*	*N*	*%*	*N*	*%*	*N*	*%*	
Sex, female		1355	44.1	3805	50.2	1157	61.9	6317	50.5	<0.001 a
Highest education level^b^										<0.001 a
	Basic	299	10.0	991	13.3	218	11.9	1508	12.3	
	Secondary	784	26.1	2093	28.1	475	26.0	3352	27.3	
	Tertiary	1922	64.0	4352	58.5	1136	62.1	7410	60.4	
		*Mean*	*SD*	*Mean*	*SD*	*Mean*	*SD*	*Mean*	*SD*	
Age		63.0	7.3	63.2	7.5	61.1	7.3	62.9	7.5	<0.001 a
Townsend deprivation index^c^		−2.02	2.57	−2.13	2.57	−1.55	2.81	−2.02	2.61	<0.001^a^

Baseline characteristics sex, age, level of education and Townsend deprivation index are shown for the three groups. Abbreviations: SD (standard deviation).

a Result is significant.

b Missing value in 2.0% of participants.

c Range in our study population was −6.3 – 9.1.

The most common trauma mechanisms in both the resilient and vulnerable group were: having been victim of a violent crime (40.1% and 38.1%, respectively), having witnessed sudden violent death (32.1% and 26.1%, respectively) and having been victim of sexual assault (26.4% and 40.7%, respectively, see Table S1, Supplementary Materials). Physical and sexual child abuse as well as physical abuse in adulthood and sexual abuse were more often experienced by women, whereas being in a life threatening accident, being victim of a violent crime or witness of a violent death were more often experienced by men (see Table S2, Supplementary Materials).

Comparing the FA of the resilient, control and vulnerable group, we found significant differences in the right CG (p ​= ​0.044, see Table 2) and in the left and right SFOF (p ​= ​0.002 and p ​< ​0.001, respectively). After adjusted Bonferroni correction, the results for the SFOF left and right remained significant.

**Table 2:** Results for the FA group comparisons using one-way ANOVA with age as a covariate.

	Mean values			ANOVA comparison	
	Resilient group	Vulnerable group	Control group	*F*	*p*
CGl	6.244∙10^−1^	6.248∙10^−1^	6.246∙10^−1^	2.361	0.094
CGr	5.826∙10^−1^	5.825∙10^−1^	5.826∙10^−1^	3.116	0.044 b
CHl	4.624∙10^−1^	4.620∙10^−1^	4.616∙10^−1^	1.133	0.322
CHr	4.626∙10^−1^	4.610∙10^−1^	4.613∙10^−1^	2.796	0.061
SFOFl	4.635∙10^−1^	4.639∙10^−1^	4.635∙10^−1^	6.030	0.002 a
SFOFr	4.619∙10^−1^	4.617∙10^−1^	4.625∙10^−1^	8.071	<0.001 a
UFl	5.139∙10^−1^	5.145∙10^−1^	5.139∙10^−1^	0.219	0.803
UFr	5.294∙10^−1^	5.279∙10^−1^	5.284∙10^−1^	2.181	0.113
CCB	7.145∙10^−1^	7.159∙10^−1^	7.150∙10^−1^	2.336	0.097
CCG	7.216∙10^−1^	7.226∙10^−1^	7.213∙10^−1^	2.950	0.052
CCS	7.911∙10^−1^	7.918∙10^−1^	7.912∙10^−1^	0.175	0.839
CCTl	5.831∙10^−1^	5.842∙10^−1^	5.836∙10^−1^	0.889	0.411
CCTr	5.517∙10^−1^	5.572∙10^−1^	5.519∙10^−1^	0.379	0.684

Mean values are shown for the three groups*, F* statistic and one-tailed *p* value are shown for the group comparisons. Abbreviations: FA (fractional anisotropy), ANOVA (analysis of variance), CG (cingulum cingulate gyrus) CH (cingulum hippocampus), SFOF (superior fronto-occipital fasciculus), UF (uncinate fasciculus), CCB (corpus callosum body), CCG (corpus callosum genu), CCS (corpus callosum splenium) and CCT (corpus callosum tapetum), l (left), r (right).

a Result is significant after correction for multiple comparisons.

b Result is significant only when uncorrected.

The mediation analysis demonstrated an effect of sex on the results for the right CG: after correction for sex, the result did not remain significant (p ​= ​0.258). The significant results for the left and right SFOF survived correction for sex (p ​= ​0.003 and p ​= ​0.001 respectively).

One-tailed T-tests were performed on the ROIs with significant results (see Table 3). The comparison between the resilient and the control group was not statistically significant in any of the ROIs. We found significantly higher FA in the left and significantly lower FA in the right SFOF in the vulnerable group compared to the control and resilient groups. We also found significantly lower FA in the vulnerable group in the right CG. Effect sizes in the significant comparisons between the resilient and vulnerable group, expressed as Cohen’s *d,* ranged from 0.053 to 0.086. For the comparisons between the vulnerable and control group, effect sizes ranged between 0.067 and 0.102.

**Table 3:** Results for the T-tests on FA for significant ROIs.

	Resilient group vs control group			Resilient group vs vulnerable group			Vulnerable group vs control group		
	*t*	*d*	*p*	*t*	*d*	*p*	*t*	*d*	*p*
CGr	0.333	0.007	0.370	1.776	0.053	0.038 a	−2.542	0.067	0.006 a
SFOFl	−0.663	0.014	0.254	−2.673	0.080	0.004 a	3.411	0.090	<0.001 a
SFOFr	−1.230	0.026	0.109	2.893	0.086	0.002 a	−3.874	0.102	<0.001 a

*t* statistic, Cohen’s *d* and one-tailed *p* value are shown for the group comparisons. In the comparison of group A vs group B, a negative *t* statistic indicates lower FA in group A. Abbreviations: FA (fractional anisotropy), ROI (region of interest), CG (cingulum cingulate gyrus), SFOF (superior fronto-occipital fasciculus), l (left), r (right).

a Result is significant.

Subsequently, MD, RD and AD means of the significant comparisons for the right CG and left and right SFOF were compared using one-tailed T-tests including the covariate age. (see Table 4). For AD, RD and MD in the left SFOF, significant differences were found between the resilient group and the vulnerable group (p ​= ​0.019, p ​= ​0.004, p ​= ​0.004, respectively). Comparing the vulnerable group to the control group in the left SFOF, RD and MD showed a significant difference (p ​= ​0.003 and p ​= ​0.007, respectively). In the right SFOF, there were significant differences between the resilient and vulnerable groups on RD and MD (p ​= ​0.002 and p ​= ​0.008, respectively). In the right SFOF, there was also a significant difference on RD and MD between the vulnerable group and the control group (p ​= ​0.005 and p ​= ​0.045, respectively).

**Table 4:** Mean values and T-test comparisons with age as a covariate for the AD, RD and MD for the resilient group vs the vulnerable group and for the vulnerable group vs the control group.

	Mean values			T test comparison			
	Resilient group	Vulnerable group	Control group	Resilient group vs vulnerable group		Vulnerable group vs control group	
				*t*	*p*	*t*	*p*
CGr							
AD	1.329∙10^−3^	1.330∙10^−3^	1.332∙10^−3^	−0.676	0.250	−1.617	0.053
RD	4.674∙10^−4^	4.679∙10^−4^	4.687∙10^−4^	−2.398	0.008 ^a^	−1.833	0.034 a
MD	7.546∙10^−4^	7.553∙10^−4^	7.565∙10^−4^	−2.289	0.011 ^a^	−0.346	0.365
SFOFl							
AD	1.164∙10^−3^	1.161∙10^−3^	1.167∙10^−3^	2.086	0.019 ^a^	−1.475	0.070
RD	5.434∙10^−4^	5.404∙10^−4^	5.447∙10^−4^	2.686	0.004 ^a^	−2.737	0.003 a
MD	7.504∙10^−4^	7.473∙10^−4^	7.522∙10^−4^	2.644	0.004 ^a^	−2.450	0.007 a
SFOFr							
AD	1.157∙10^−3^	1.153∙10^−3^	1.160∙10^−3^	1.064	0.144	−0.300	0.382
RD	5.336∙10^−4^	5.314∙10^−4^	5.348∙10^−4^	2.856	0.002 ^a^	−2.582	0.005 a
MD	7.413∙10^−4^	7.385∙10^−4^	7.433∙10^−4^	2.442	0.008 ^a^	−1.700	0.045 a

Mean values are shown for the three groups, *t* statistic and one-tailed *p* value are shown for the group comparisons. Abbreviations: CG (cingulum cingulate gyrus), SFOF (superior fronto-occipital fasciculus), l (left), r (right), AD (axial diffusivity), RD (radial diffusivity), MD (mean diffusivity).

a Result is significant.

Finally, in the post hoc analyses on childhood maltreatment, the resilient and vulnerable groups consisted of 625 and 525 participants, respectively, in the wider definition of childhood maltreatment, and of 85 and 121 participants, respectively, in the narrower definition. In both analyses, age was added as a covariate. In the first analysis, the one-way ANOVA was significant in the left and right CG, left CH, left and right SFOF, right UF and CCG. The results for the left and right SFOF survived correction for multiple testing. The post hoc T-tests did not demonstrate a significant difference between the resilient and the control group in any of the ROIs. In the second analysis (narrower definition), the one-way ANOVA showed significant differences in the right CG, right CH and CCG only when uncorrected for multiple testing. Explorative post hoc T-tests for this result showed a significant difference in the right CG and right CH between the resilient and control group (p ​= ​0.048 and p ​= ​0.003, respectively) as well as between the resilient and vulnerable group (p ​= ​0.007 and p ​= ​0.008, respectively).

## 4 Discussion

This study sought to identify white matter correlates of resilience in a large population sample of 40-69-year-old volunteers from the UK Biobank. We compared three groups of subjects; a resilient, vulnerable and control group. We did not find white matter correlates of resilience in the main analyses, nor in our post-hoc sensitivity analysis in childhood maltreatment subgroups.

There are several possible explanations for the absence of a clear association of white matter connectivity with resilience in the selected ROIs. Since mean tract values were used we could have missed an effect that only voxelwise analyses would have detected. Secondly, the UK Biobank study population is reflective of a general, middle-aged population, which is in contrast with populations of carefully selected and well-trained professionals such as first-responders typically included in most studies on resilience. Opting for a broader population sample also necessitated expanding the definition of trauma that was used. Including, for example, domestic abuse, as well as life-threatening accidents and witnessing violent death as traumatic events, resulted in the inclusion of a variety of traumatic experiences, as shown in Table S1. As some studies suggest that different traumatic experiences could each have distinctive biological signatures, one may argue that lumping these traumatic experiences together might have reduced the power. Our post-hoc analysis in a population with a more homogeneous definition of trauma, however, also revealed no specific correlates of resilience to more severe forms of childhood maltreatment in subjects from this general population sample.

Essentially, especially regarding the large sample size of this study, our results indicate that the regions of interest of this study are not correlated with resilience in the general, middle-aged population.

We found abnormalities in our vulnerable group compared to the control and resilient group. There was increased mean FA in the left and decreased mean FA in the right SFOF ROI. Higher FA is associated with greater white matter integrity, specifically, greater axon diameter, density or myelination (Beaulieu, 2002). Our findings for the left and right SFOF, a tract associated with attention and vigilance, may represent a vulnerability marker or a consequence of the various sequelae of the exposure to a traumatic event (Milner and Goodale, 2008; Wakana et al., 2004). The study by Aschbacher et al. (2018) and the ENIGMA PTSD study by Dennis et al. (2019) contradict each other on the results for the SFOF. While taking into account that both studies are quite different from our study regarding design and inclusion criteria, our findings for the SFOF are not easy to interpret. From our results, it also seems that the effect of vulnerability on MD and RD differs between white matter tracts. More investigation is required to interpret the combinations of increased and decreased MD and RD in the context of resilience and vulnerability.

We believe that the present study has several strengths. It is the largest to date to investigate white matter correlates of resilience. We were also able to investigate the effect of age and sex in relation to our outcome variables. In comparison to most other studies, we included a healthy, nonexposed control group and expanded our definition of trauma-related psychopathology to include mental disorders other than PTSD, in particular affective disorders, to obtain a more accurate picture of vulnerability and resilience. Finally, utilization of the adapted Bonferroni correction ensured correction for multiple testing tailored to our intercorrelated outcome variables.

There are some limitations to report as well. The retrospective nature of the UK Biobank questionnaires we used for in-and exclusion and group selection introduced a risk of recall bias. Furthermore, in the absence of longitudinal data, our study was not designed to make causal inferences, nor could it deduce whether white matter differences were the result of an acquired resilience or vulnerability, or if such differences were present in the participants prior to trauma exposure. Unfortunately, no data was available on the time lapse between trauma exposure and inclusion in the study, or whether this differed between the resilient and vulnerable groups. Information on whether the development of a participant’s mental health disorder was associated with the participant’s traumatic experience was also absent, which might have decreased the quality of the vulnerable group. As mentioned previously, the utilization of mean tract values instead of voxelwise data might have obscured an association with resilience. Out of necessity, our ROI selection was based on limited literature. Possibly, other white matter tracts could be implicated in resilience. Lastly, only white matter tracts were investigated in this study. Previous studies have identified correlates of resilience using other morphometric and functional magnetic resonance imaging approaches (van der Werff et al., 2013).

In conclusion, our results could suggest that there are no specific white correlates of resilience in the general population. Clearly, this should be corroborated in future studies which should ideally focus on collecting longitudinal data as well as using validated questionnaires and patient records to minimize risk of recall bias. We also recommend that future studies on white matter correlates of resilience conduct voxelwise analyses if possible, to improve sensitivity, and investigate other white matter tracts that might be implicated in resilience.

## Declaration of competing interest

All authors declare that they have no conflicts of interest.
