# Supplementary material for: White matter connectivity in resilience in a general population sample of 12,516 individuals
Source: Neurosci Appl. 2023 Jul 27;2:101130. doi: 10.1016/j.nsa.2023.101130 (PMC12244105; doi:10.1016/j.nsa.2023.101130)
Supplement: Multimedia component 1 [file mmc1.docx]

|  | Resilient (N=3070) | | Vulnerable (N=1771) | | Total (N=4841) | |
| --- | --- | --- | --- | --- | --- | --- |
|  | *N* | *%* | *N* | *%* | *N* | *%* |
| Physical child abuse | 475 | 15.5 | 400 | 21.4 | 875 | 18.1 |
| Sexual child abuse | 199 | 6.5 | 211 | 11.3 | 410 | 8.5 |
| Physical abuse in adulthood | 295 | 9.6 | 345 | 18.5 | 640 | 13.2 |
| Life-threatening accident | 604 | 19.7 | 333 | 17.8 | 937 | 19.4 |
| Victim of violent crime | 1230 | 40.1 | 713 | 38.1 | 1943 | 40.1 |
| Witness of violent death | 984 | 32.1 | 487 | 26.1 | 1471 | 30.4 |
| Sexual assault | 811 | 26.4 | 761 | 40.7 | 1572 | 32.5 |

**Table S1: Trauma mechanisms for the resilient and vulnerable group**
Prevalence of different trauma mechanisms are shown for the resilient and vulnerable group.

**Table S2: Trauma mechanisms by sex**

|  | Female (N=6317) | | Male (N=6199) | | *p* |
| --- | --- | --- | --- | --- | --- |
|  | *N* | *%* | *N* | *%* |  |
| Physical child abuse | 477 | 54.5 | 398 | 45.5 | 0.013 |
| Sexual child abuse | 324 | 79.0 | 86 | 21.0 | <0.001 |
| Physical abuse in adulthood | 509 | 79.5 | 131 | 20.5 | <0.001 |
| Life-threatening accident | 356 | 37.7 | 588 | 62.3 | <0.001 |
| Victim of violent crime | 758 | 38.8 | 1195 | 61.2 | <0.001 |
| Witness of violent death | 539 | 36.5 | 936 | 63.5 | <0.001 |
| Sexual assault | 1194 | 75.6 | 386 | 24.4 | <0.001 |

Prevalence of different trauma mechanisms are shown for both sexes. Statistical analysis was performed using a chi square test.
